# Patient-Derived Explants of Colorectal Cancer: Histopathological and Molecular Analysis of Long-Term Cultures

**DOI:** 10.3390/cancers13184695

**Published:** 2021-09-19

**Authors:** Sara da Mata, Teresa Franchi-Mendes, Sofia Abreu, Bruno Filipe, Sónia Morgado, Marta Mesquita, Cristina Albuquerque, Ricardo Fonseca, Vítor E. Santo, Erwin R. Boghaert, Isadora Rosa, Catarina Brito

**Affiliations:** 1 Serviço de Anatomia Patológica, Instituto Português de Oncologia de Lisboa Francisco Gentil (IPOLFG, EPE), Rua Prof. Lima Basto, 1099-023 Lisboa, Portugal; smata@ipolisboa.min-saude.pt (S.d.M.); smorgado@ipolisboa.min-saude.pt (S.M.); mmesquita@ipolisboa.min-saude.pt (M.M.); rifonseca@ipolisboa.min-saude.pt (R.F.); 2 NOVA Medical School, Universidade Nova de Lisboa, Campo dos Mártires da Pátria 130, 1169-056 Lisboa, Portugal; 3 Instituto de Biologia Experimental e Tecnológica, Apartado 12, 2780-901 Oeiras, Portugal; mtmendes@ibet.pt (T.F.-M.); sofia_abreu5@hotmail.com (S.A.); vitorespsanto@gmail.com (V.E.S.); 4 Instituto de Tecnologia Química e Biológica António Xavier, Universidade Nova de Lisboa, Av. da República, 2780-157 Oeiras, Portugal; 5 Unidade de Investigação em Patobiologia Molecular (UIPM), Instituto Português de Oncologia de Lisboa Francisco Gentil (IPOLFG, EPE), Rua Prof. Lima Basto, 1099-023 Lisboa, Portugal; bfilipe@ipolisboa.min-saude.pt (B.F.); calbuque@ipolisboa.min-saude.pt (C.A.); 6 Faculdade de Medicina da Universidade de Lisboa, Avenida Prof. Egas Moniz MB, 1649-028 Lisboa, Portugal; 7 Abbvie Inc., 1 North Waukegan Road, North Chicago, IL 60064-6098, USA; erwin.boghaert@abbvie.com; 8 Serviço de Gastrenterologia, Instituto Português de Oncologia de Lisboa Francisco Gentil (IPOLFG, EPE), Rua Prof. Lima Basto, 1099-023 Lisboa, Portugal; 9 The Discoveries Centre for Regenerative and Precision Medicine, Lisbon Campus, Av. da República, 2780-157 Oeiras, Portugal

**Keywords:** colorectal cancer, patient-derived explants, translational models

## Abstract

**Simple Summary:**

Colorectal cancer is the third most common cancer type among men and women. Prescription of medical treatments for cancer often relies on a process of trial and potential error, more recently guided by patient stratification based on biomarkers. Nonetheless, available biomarkers do not accurately predict patient response and there is a need for predictive and translational models to provide proper clinical information on treatment guidance. Herein, we developed an ex vivo model of colorectal cancer, using fresh tumour samples to establish explant cultures, taking advantage of agitation-based culture systems. We performed a thorough characterisation over one month in culture and observed preservation of original tumour genetic features and partial preservation of architecture and non-malignant cells that compose the tumour microenvironment. Our findings highlight the importance of detailed model characterisation and support the applicability of our model in pre- and co-clinical settings.

**Abstract:**

Colorectal cancer (CRC) is one of the most common cancers worldwide. Although short-term cultures of tumour sections and xenotransplants have been used to determine drug efficacy, the results frequently fail to confer clinically useful information. Biomarker discovery has changed the paradigm for advanced CRC, though the presence of a biomarker does not necessarily translate into therapeutic success. To improve clinical outcomes, translational models predictive of drug response are needed. We describe a simple method for the fast establishment of CRC patient-derived explant (CRC-PDE) cultures from different carcinogenesis pathways, employing agitation-based platforms. A total of 26 CRC-PDE were established and a subset was evaluated for viability (*n* = 23), morphology and genetic key alterations (*n* = 21). CRC-PDE retained partial tumor glandular architecture and microenvironment features were partially lost over 4 weeks of culture. Key proteins (p53 and Mismatch repair) and oncogenic driver mutations of the original tumours were sustained throughout the culture. Drug challenge (*n* = 5) revealed differential drug response from distinct CRC-PDE cases. These findings suggest an adequate representation of the original tumour and highlight the importance of detailed model characterisation. The preservation of key aspects of the CRC microenvironment and genetics supports CRC-PDE potential applicability in pre- and co-clinical settings, as long as temporal dynamics are considered.

## 1. Introduction

Colorectal cancer (CRC) is one of the most common cancers worldwide [1,2]. It is a heterogeneous entity that derives from different pathways with clinical impact [3]. These pathways include microsatellite instability (MSI), chromosomal instability (CIN) and CpG island hypermethylation (CIMP) [3]. CRC can exhibit simultaneous features of several pathways—e.g., about 25% of MSI tumours can exhibit CIN and most of them also present CIMP [4]. CRC heterogeneity, amongst patients and within the same tumour, contributes to drug failure and relapse [1,5]. CRC molecular subtype is associated with drug resistance—MSI is linked with resistance to 5-Fluorouracil (5-FU) and CIMP may also imply a lack of benefit from this therapy [4], while *KRAS* mutations confer resistance to cetuximab [6,7].

The importance of tumour microenvironment (TME) in CRC progression is widely recognised [8]—drug resistance and drug targeting TME elements have been reported [9]. Examples of this TME relevance are the association between intraepithelial lymphocytes and MSI-CRC [10], which seem to correlate with a more favourable outcome [11] and the positive prognostic value of a high frequency of tumour infiltrating regulatory T cells in Mismatch repair (MMR) proficient CRC [12]. Cancer-associated fibroblasts and M2 macrophages also seem to correlate with clinical outcome [9,13], while a higher microvessel density and endothelial cells secreted factors could be involved in therapeutic resistance [9,14].

In the clinical setting, the prescription of medical treatments for cancer often relies on a process of trial and potential error, more recently guided by patient stratification based on biomarkers [15]. However, this approach still does not capture the complexity associated with intratumoral heterogeneity, both hierarchical and stochastic, in the variability of chemotherapy response [16]. Improving CRC pre-clinical models is essential to contribute to personalised treatment and therapeutic response prediction. Current drug testing and screening rely on 2D cell lines and patient-derived xenografts (PDX), both with recognised limitations [17]. Most cancer cell lines are selected subpopulations and do not represent the architecture, physiology or progression of the native tumour [18,19]. PDX in immunocompromised mice may capture features of tumour heterogeneity, but they lack immune system interactions [17,19] and the TME is progressively replaced by host cells [20]. Moreover, the establishment of PDX is cost and time-intensive, which may hinder its clinical application [19,21]. There is a clear need for representative cancer 3D models that can recapitulate the TME more faithfully than monolayer cultures [17]. 3D cell cultures have been a growing approach to mimic CRC [8] and among the most applied methods is the use of multicellular tumour spheroids; other attempts include the use of scaffolds, natural (such as Matrigel) or synthetic [8,21]. Patient-derived organoids recapitulate the genetic properties of the original tumour, though they still lack cancer-associated stroma [22,23,24,25]. Patient-derived ex vivo models are promising approaches for preservation of the tumour and its native microenvironment [20,26]. Amongst these, patient-derived explants (PDE) and more recently ultrathin tissue slices and have been explored [27,28,29,30]. The short-term duration of these ex vivo models, typically up to 72 h, hinders cyclic drug exposure regimens and evaluation of resistance mechanisms [31]. Moreover, most reports focus on drug response effects and readouts of cell death, and do not characterise thoroughly the model at a baseline level [32]. Recently, we hypothesised that larger PDE could have advantages over thin slices by the higher representation of the tumour microenvironment components, contributing to sustain heterotypic cellular crosstalk within the tissue and improving its longevity in culture; we reasoned that dynamic culture would improve cell viability and phenotype ex vivo, by guaranteeing efficient diffusion of oxygen and soluble compounds. Recently, we successfully employed this rational to OvC-PDE [33], and here we tested its applicability to CRC tissue.

In this work, we developed a patient-derived long-term, reproducible PDE model of CRC (CRC-PDE), with the main objective of providing a more clinically relevant system to address issues presented by precision medicine approaches. We show that CRC-PDE retain key molecular and histological features of the parental tumours. Despite some degree of morphological rearrangement and a decrease in cellularity, the TME components are partially preserved in the CRC-PDE model.

## 2. Materials and Methods

### 2.1. Prospective Study of Ex Vivo Cultures of CRC-PDE

Study approval was performed by the clinical institution’s Review Board and Ethics Committee and informed consent was obtained from the patients. Consecutive patients, older than 18 years old, with CRC proposed for primary tumour surgery without neoadjuvant therapy by Instituto Português de Oncologia de Lisboa, Francisco Gentil (IPOLFG)’s Colorectal Cancer Multidisciplinary Group were selected for the study. Patients were excluded if informed consent could not be obtained or if they were human immunodeficiency virus (HIV), hepatitis B virus (HBV) or hepatitis C virus (HCV) positive, due to the research lab’s safety policy.

### 2.2. Tumour Tissue Collection and Processing

Fresh tumours were collected from patients at the time of surgery at IPOLFG. Tumour samples were processed as recently published by our team for ovarian carcinoma samples [33]. Briefly, tumour specimens were transported in Dulbecco’s Modified Eagle Medium: Nutrient Mixture F-12 (DMEM/F12, Gibco) and processed within 4 h. Samples were washed with Dulbecco’s Phosphate Buffered Saline (DPBS, Gibco), weighted and mechanically dissociated into fragments of ~1 mm^3^.

### 2.3. Establishment of CRC-PDE Cultures

In total, 100 tumour fragments of ~1 mm^2^ (colorectal cancer patient-derived explants, CRC-PDE) were transferred to 125 mL shake flasks (Erlenmeyer, Corning) with 20 mL of DMEM/F12, supplemented with Primocin (ThermoFisher), B27 (ThermoFisher), Gastrin I (Sigma), Prostaglandin (Sigma), Nicotinamide (Sigma), N-acetylcysteine (Sigma) and EGF (ThermoFisher) [34,35], to generate cultures at 5 CRC-PDE/mL. Cultures were kept under orbital shaking (IKA KS 260 basic) at 100 rpm, in an incubator (Nuaire US Autoflow) at 37 °C, 5% CO_2_ in air. CRC-PDE were sampled at day 0 (surgery day), and each week afterwards for the total duration of the culture. CRC-PDE cultures were dependent on initial sample size and culture expenditure due to characterisation based on destructive endpoints. Therefore, different CRC-PDE were terminated at distinct timepoints and not all CRC-PDE cultures could be evaluated for all readouts.

### 2.4. Surface Area and Concentration of CRC-PDE

CRC-PDE surface area and concentration were quantified at each timepoint. In total, 1–2 mL of CRC-PDE culture were collected and observed by phase-contrast microscopy (DMI6000 Leica Microsystems CmBH, Wetzlar, Germany). Size measurements were performed using open-access Image J 1.53c Software [36,37]. A threshold was applied to generate binary images and automatic analysis was done applying the Analyse Particles function and extracting the area measurements (mm^2^). Explant concentration was determined as the number of explants per mL of culture.

### 2.5. Cell Viability and Histological Characterisation of CRC-PDE

Viability was analysed by live/dead assay, resazurin reduction capacity and tissue observation. Live/dead assay was performed as described previously [33]. Briefly, fluorescein diacetate (FDA, 10 µg/mL in DPBS, Molecular Probes) and propidium iodide (PI, 2 µg/mL in DPBS, Molecular Probes) were used to label live and dead cells, respectively. At each time point, 3–5 explants were collected, stained with FDA and PI, and visualised using a fluorescence microscope (DMI6000 Leica Microsystems CmBH, Wetzlar, Germany). Image analysis was performed with Image J Software.

Resazurin reduction capacity was evaluated using the PrestoBlue Cell Viability Reagent (A13262, Invitrogen), as described before [33]. At each time point, 1 mL of culture suspension (on average, 5 PDE) was collected in triplicates, and incubated with PrestoBlue reagent (diluted 1:10) for 1 h at 37 °C. After this, supernatants were collected to a 96-well black fluorescence reading plate (Corning) and fluorescence reading was performed (Infinite 200 PRO NanoQuant plate reader, TECAN).

Cell viability was also documented by brightfield microscopy observation of Hematoxylin and Eosin (HE) stained tissue (Hematoxylin, Cat. Number CS700, Dako; and Eosin, Cat. Number CS701, Dako). For each time point, at least 1 mL of the sample was fixed in formol, embedded in paraffin, and stained with HE. At the end of each culture, all explants were collected for a final evaluation. HE stained samples were evaluated for gland density, tumour cell senescence, stroma cellularity and inflammatory cell density using a semiquantitative manual (eyeballing) system, in 33% intervals. The reference value was the status of each variable at day 0 (day of surgery) representing the phenotype and morphology of the original tumour and for each time point of evaluation, the proportion of each variable was compared to it. Gland density, stroma cellularity and inflammatory cell density were scored 1 at day 0, while tumour senescence was scored 0. Glands were defined as cohesive structures of epithelial cells surrounding a lumen/space; stroma cells as mesenchymal cells in the stroma compartment; inflammatory cells as mature leukocytes in the stroma (including lymphocytes, neutrophils, eosinophils, mast cells and macrophages). The tumour senescent phenotype was defined by cell morphological change with increasingly eosinophilic cytoplasm, flat shape, and vacuolisation [38,39]. The presence or absence of capillaries was also documented. CRC-PDE and their original counterparts were also assessed for architecture, p53 immunohistochemical expression (Cellmarque 453M-85) and 4 MMRp immunohistochemical expression (MLH1: Ventana 490-7535, MSH2: Dako IR085, MSH6: Dako IR086, PMS2: Dako IR087). Expression of p53 was deemed overexpressed when at least 50% of tumour cells showed nuclear staining, absent when there was no nuclear staining and wild type otherwise, in keeping with this marker correlation to TP53 mutational status [40]. Image processing was performed with Aperio ImageScope software (v12.3.3.5048, Leica).

### 2.6. Molecular Characterisation of CRC-PDE

Genomic DNA was extracted from formaldehyde-fixed paraffin-embedded CRC, normal mucosa and CRC-PDE samples using the KAPA Express Extract Kit (KAPABIOSYSTEMS). The MSI status was analysed using Bethesda microsatellite markers: BAT26, D17S250, D2S123, BAT25 and D5S346 [41,42]. Each tumour and paired normal DNA was amplified for all markers by PCR, using fluorescently labelled primers (Applied Biosystems, Foster City, CA, USA), specific for each locus, as previously described [43]. PCR products were analysed in the ABI Prism 3130 genetic analyser using the GeneMapper software, version 5.1 (Applied Biosystems). In tumour samples exhibiting MSI in only one marker, or without a conclusive result in at least one marker, two additional markers were analysed (BAT40 and MYCL1). Tumours presenting MSI in two or more microsatellite markers were classified as MSI-High (MSI-H), whereas MSI-Low (MSI-L) was defined by the presence of MSI only in one of the respective markers. Tumours without MSI in any of the markers were considered microsatellite stable (MSS). The mutational status of *KRAS* exon 2, codons 12 and 13, was detected by qPCR with Idylla™ KRAS Mutation Test (Biocartis, CEIVD). For selected cases, DNA from samples of tumour tissue was amplified by PCR using primers for *KRAS* exon 2 and *BRAF* exon 15 and the product was sequenced using Sanger sequencing on Big Dye terminator v1.1 sequencing kit (Applied Biosystems) on an automatic ABI PrismTM 3130 Genetic Analyzer (Applied Biosystems).

### 2.7. Cytotoxic Drug Assays

CRC-PDE from 6 distinct tumours (CRC5, CRC22, CRC23, CRC24, CRC25 and CRC26) were distributed into 12-well plates (5–10 PDE/well, in triplicates) and cultured under orbital shaking. These CRC-PDE were challenged with 100 µg/mL of 5-Fluorouracil (5-FU), for two weeks; for 3 cases (CRC5, CRC21 and CRC22), cultures were also challenged with the combination of 100 µg/mL of 5-FU and 3.2 µg/mL of Oxaliplatin (OXA). Drug concentrations were chosen based on previous reports [44]. Drug challenge started at day 4 of culture (or at day 7 for CRC5-PDE) and the drugs were replenished daily; cultures were sampled after 1 and 2 weeks of drug exposure. Cell viability was evaluated by morphology (HE observation) and cell death by the leakage of lactate dehydrogenase (LDH) into the culture medium (LDH_CM_). Drug-induced cell death was determined by subtracting LDH_CM, vehicle control_ to LDH_CM, drug_, relative to LDH_total_ (LDH_CM, vehicle control_ + LDH_lysate, vehicle control_). The PDE lysate was obtained by incubation of at least one vehicle control well with 10% TritonX-100, overnight, to release all LDH content. LDH was determined using the commercial kit Pierce LDH Cytotoxicity Assay (ThermoFisher).

### 2.8. Statistical Analysis

Data are shown as mean ± standard deviation (SD) of N (indicated in each figure legend). Statistical and data analysis were carried out using GraphPad Prism 9.0.0 software for Windows (GraphPad Software, La Jolla California USA, www.graphpad.com accessed date 31 August 2021), as indicated in each figure legend.

## 3. Results

### 3.1. Viability and Histological Characterisation of CRC-PDE Cultures

We have recently reported a method for the generation of PDE from ovarian carcinoma by mechanical dissociation of tumour tissue into fragments of approximately 1 mm^3^ and their culture under orbital agitation [33]. Here, we adapted this methodology to establish PDE cultures from surgically resected CRC parental tissue. Samples from a total of 26 CRC were collected. Patient clinicopathological data are summarised in Table 1 and detailed in Table S1.

Tumour samples were processed as described in the methods section and cultured as CRC-PDE for 7–122 days (median, 28 days). For 92% of the cultures, the limiting factor for culture duration was the CRC-PDE low number due to sequential sampling (*N* = 24/26). One culture was terminated by the second week due to fungal contamination and another one due to low cell viability.

**Figure 1:**
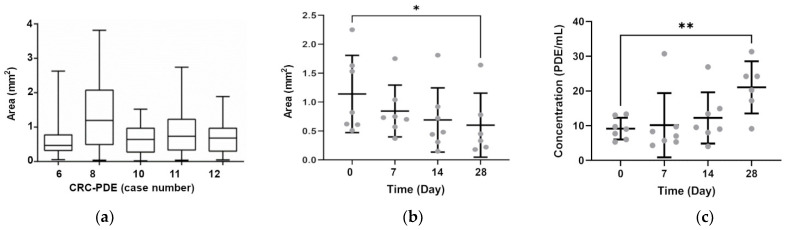
CRC-PDE area and concentration. (**a**) Measurement of CRC-PDE area after tumour sample processing (day 0); box-and-whisker plot shows the median (middle lines), interquartile range (boxes) and minimum to maximum values (whiskers); data were calculated from measurements of at least 15 explants. (**b**) CRC-PDE area and (**c**) CRC-PDE concentration, along 4 weeks of culture; data are presented as mean ± SD, calculated from at least seven biological replicates; statistical significance was evaluated by a mixed effects model for analysis of repeated measurements followed by post-hoc Dunnett’s test comparing with day 0; * *p* < 0.05, ** *p* < 0.01. CRC-PDE, colorectal cancer patient-derived explants.

CRC-PDE cultures derived from 23 tumours were characterised along the first 4 weeks of cultures, with the evaluation of cell viability, tumour morphology, stroma constituents and immunohistochemical and molecular features. Immediately after sample processing (day 0), the average area of CRC-PDE was 1.0 ± 0.9 mm^2^ (Figure 1a).

**Figure 2:**
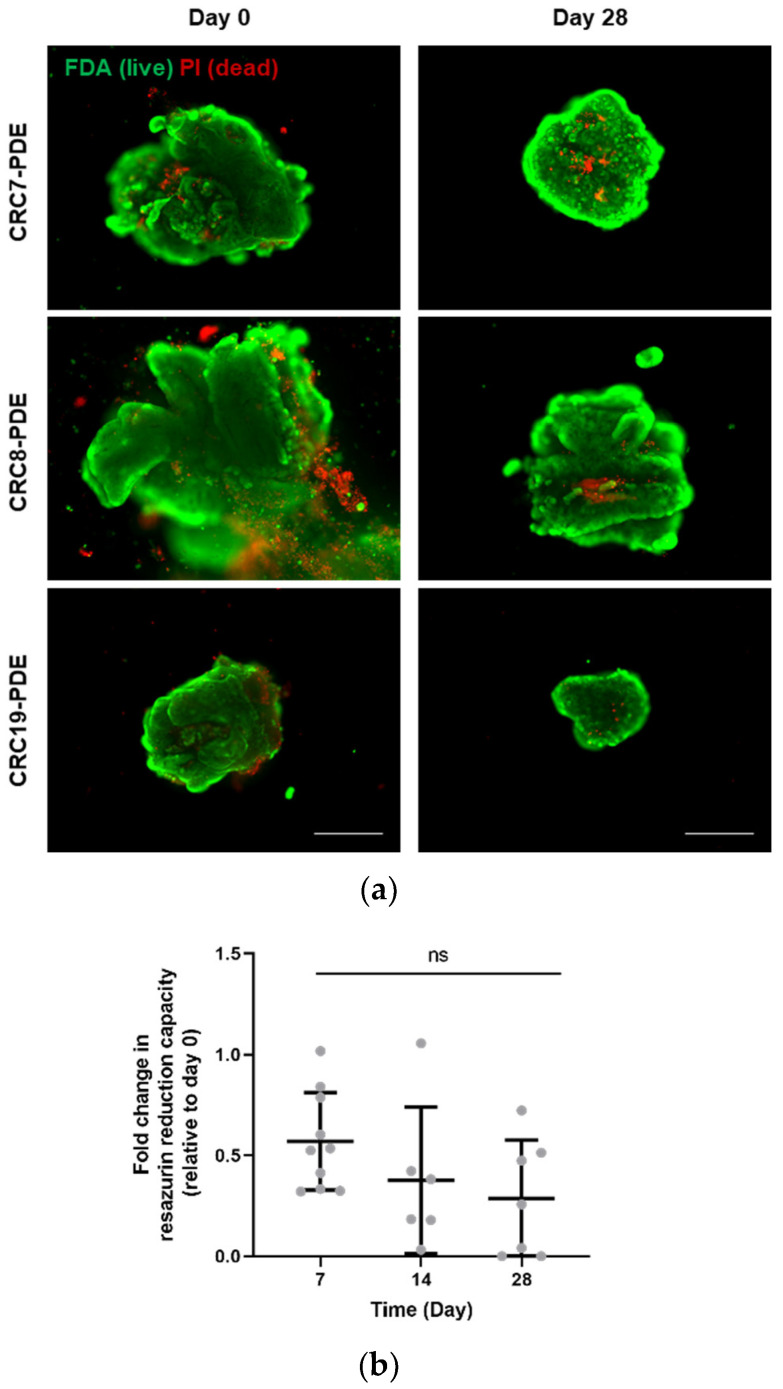
CRC-PDE culture cell viability. (**a**) Representative images of CRC-PDE culture at day 0 (immediately after processing of the tumour tissue) and after 4 weeks, stained with fluorescein diacetate (FDA, green) and Propidium Iodide (PI, red) for detection of live and dead cells, respectively. Scale bar: 500 µm. (**b**) Measurement of resazurin reduction capacity of the CRC-PDE cultures along time. Data are presented as the mean of fold change relatively to day 0 ± SD, calculated from 10 biological replicates. Statistical significance was evaluated by a mixed effects model for analysis of repeated measurements followed by a post-hoc Tukey’s multicomparison test. CRC-PDE, colorectal cancer patient-derived explants; ns, non-significant.

During culture, the PDE average size gradually decreased from 1.1 ± 0.7 mm^2^ at day 0 to 0.8 ± 0.4 mm^2^ and 0.6 ± 0.5 mm^2^ at days 7 and 28, respectively (Figure 1b). In parallel, we detected an increase in PDE concentration, with 9.1 ± 3.1 PDE/mL at day 0 and 21.0 ± 7.5 PDE/mL by day 28 (Figure 1c). This concomitant size reduction and concentration increase were probably due to PDE fragmentation. Despite the size decrease, CRC-PDE retained high cell viability during culture, as observed by a fluorescent live/dead assay that assesses cell membrane integrity. At day 0, viability was high but a few regions with dead cells were typically observed in the explant periphery (Figure 2a), which could be a consequence of the mechanical processing. In fact, CRC-PDE metabolic activity, measured by resazurin reduction capacity, showed a decrease of approximately 50% during the first week of culture but remained relatively stable throughout the later time points (Figure 2b). Despite the initial cell death, the results suggest an adaptation to the in vitro setting and cell viability could be maintained throughout the remaining culture period.

**Figure 3:**
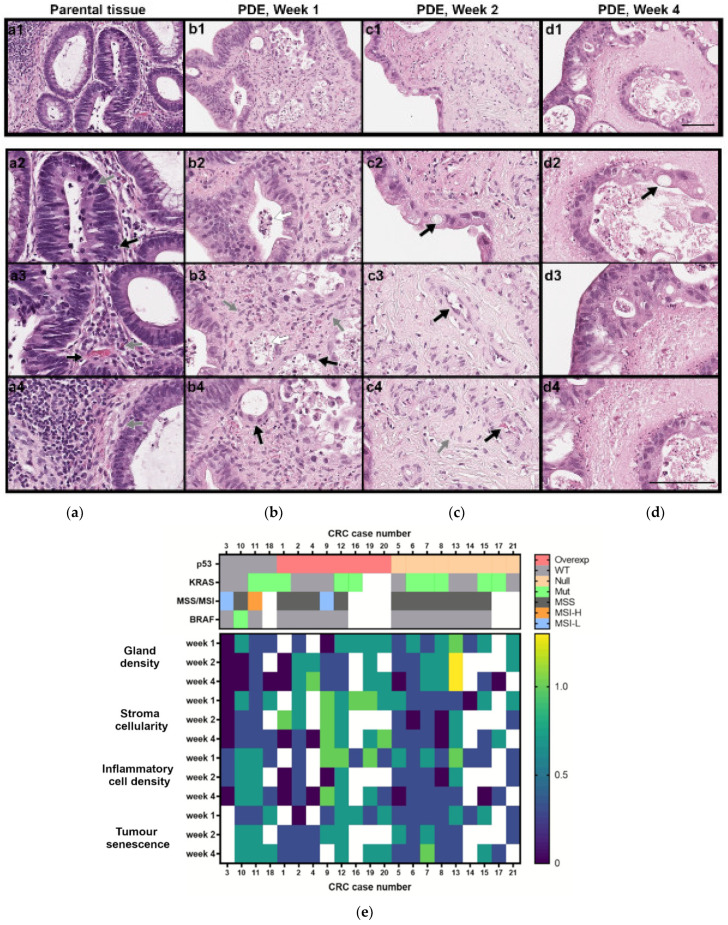
Histopathological and molecular characterisation of CRC-PDE cultures. (**a**–**d**) Representative HE images of the parental tumour and derived explants (PDE) over 4 weeks of culture (for case CRC2). Gland-forming tumour cells (**a1**–**a3**,**b2**,**d3**), mitotic cells (**a2**, grey arrow), vascular stroma (**a3**,**c3**, black arrows), stromal fibroblast (**a4**,**b3**,**c4**, grey arrows), tumour infiltrating lymphocytes (**a2**, black arrow) and plasma cells (**a3**, grey arrow), erythrocytes (**c4**, black arrow), senescent cells (**b3**,**b4**,**c2**,**d2**, black arrows), necrotic cells (**b2**,**b3** white arrows), acellular stroma (**d4**). Scale bars, 100 µm. (**e**) Heatmap of the histopathological features along culture time (week 1—days 7–9; week 2—days 14–17; week 3: days 25–28) and mutation and protein alterations associated with CRC carcinogenesis, for the 21 CRC cases analysed. Scores for morphological features were performed considering day 0 of culture as the reference and scored as 1 for gland density, stroma cellularity and inflammatory cell density and as 0 for senescence evaluations. White boxes indicate not determined. CRC, colorectal cancer; CRC-PDE, colorectal cancer patient-derived explants; MSS, microsatellite stable; MSI-L, microsatellite instability-low; MSI-H, microsatellite instability-high; Mut, mutated; Overexp, overexpressed; WT, wild type.

Histopathological characterisation of CRC-PDE derived from 23 tumours was performed, and HE analysis showed that most CRC-PDE cultures had viable tumour cells for the entire culture duration (96%; *N* = 22/23, Figure 3a–e and Figure S1a–d).

All original tumours evaluated were gland forming (Figure 3(a1–a3)), had mitotic cells (Figure 3(a2), grey arrow) and capillary structures were observed (Figure 3(a3), black arrow), as well as cellular stroma rich in fibroblasts (Figure 3(a4), grey arrow) and different degrees of immune cells, such as tumour infiltrating lymphocytes (Figure 3(a2), black arrow) and plasma cells (Figure 3(a3), grey arrow). During culture time, viable stroma cells were observed as long as neoplastic cells were present (87%; *N* = 20/23, Figure 3a–d). Simultaneously, a variable amount of necrosis was present in CRC-PDE (Figure 3(b2,b3), white arrows). In most of the CRC-PDE cultures, tumour glandular architecture (71%, *N* = 15/21, Figure 3(b2,b3)) and inflammatory cells in the stroma (76%, *N* = 16/21) were retained throughout the first 4 weeks (Figure 3a–d). The gland density, stroma and immune cells diminished up to the second week of culture but remained steady for the remaining time (Figure 3(b3,c4,d4) and Figure S1a–d), with the identification of T lymphocytes (CD3 positive cells) and macrophages (CD68 positive cells) along culture time (Figure S2a). Tumour cells progressively gained a senescent phenotype in the first two weeks of culture and had fewer mitoses, without further accentuation of this phenotype thereafter (Figure 3(b3,b4,c2,d2), black arrows; Figure S1d). Towards the end of the culture, tumour cells were predominantly located at the periphery of the CRC-PDE (Figure 3(c2,d3)). At week 4, the vascular network of CRC was absent in 91% (19/21) of the explants.

### 3.2. CRC-PDE Representation of the Original Tumours

All original tumours included in this analysis (*N* = 21) extensively formed glandular structures (low-grade tumours). The expression of p53 in the invasive parental tumours was homogenous in 95% of the cases (*N* = 20/21), with overexpressed (*N* = 8), absent (*N* = 8) or wild type (*N* = 4) expression patterns (Figure S1f). MMR protein detection was retained in all original tumours except for CRC11, which lost PMS1 homolog 2, mismatch repair system component (PMS2) expression. *KRAS* mutations were found in 50% of the parental tumours (*N* = 8/16), *BRAF* V600E was present in 7% (*N* = 1/15) and MSI in 20% (*N* = 3/15) (Figure 3e).

In general, CRC-PDE retained glandular architecture and immunohistochemical and genetic features of their parental counterparts (95%, *N* = 20/21 Figure 3, Table S1). For one of the cultures (CRC1), *KRAS* mutational status was consistently different from the original counterpart. *KRAS* status was evaluated on four different areas of the original tumour, including the mirror sample used for PDE culture and a lymph node metastasis, with the same result (no mutation). On the other hand, the tumoral area, from which the CRC1-PDE was generated, had a c.35G>A, p.Gly12Asp mutation, that was consistently detected at day 0 and during culture. CRC1 tumour was also the only case with heterogeneous p53 staining. The tumour had areas of wild type and areas of mutant *TP53* (IHC staining for p53 as a surrogate of *TP53* mutational status). In PDE culture, this heterogeneity was retained (Figure S3). CRC13 had no p53 expression on the invasive tumour and a wild type expression pattern on the dysplastic pre-invasive lesion (an adenoma). PDE retained both patterns of expression (p53 absent type—day 0 sample; p53 absent and wild type—week 1 sample; p53 wild type—week 4 sample), suggesting that both invasive and pre-invasive lesions were preserved in culture. The evaluation of the mutated allele peak at the Sanger sequencing electropherogram relative to the wild-type allele peak showed that for 4 of 10 CRC-PDE with *KRAS* mutation, the mutated cells seemed to be positively selected over time; by week 4 of culture, there were 50% more mutated clones in the culture relative to day 0. The reverse was observed for the CRC15-PDE (evaluation at day 0 and week 1).

### 3.3. Challenge of CRC-PDE Cultures with Cytotoxic Drugs

**Figure 4:**
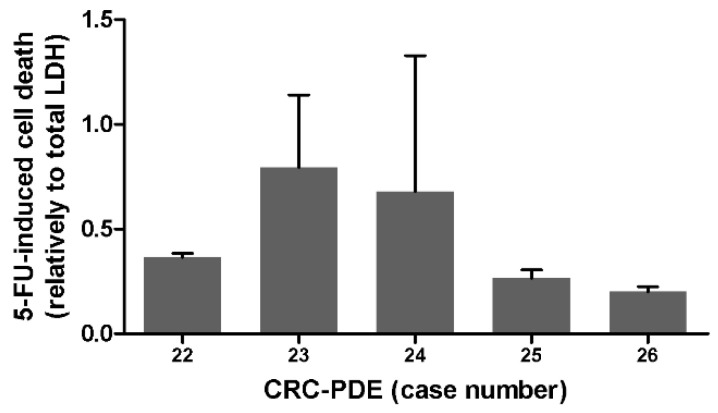
Drug challenge in CRC-PDE cultures. Drug-induced cell death determined by LDH leakage relative to total LDH content, in different CRC-PDE cultures, after 2 weeks of drug exposure. 5-FU 5-fluorouracil, CRC-PDE colorectal cancer patient-derived explant culture.

As a proof of concept for drug susceptibility tests, CRC-PDE cultures derived from five distinct cases were challenged with the standard of care drug for CRC, 5-FU. HE analysis was performed for three of the cultures; in the control samples of each CRC-PDE tumour and stroma cell viability was maintained (*N* = 3/3), whereas in cultures exposed to 5-FU no viable tumour cells were detected and viable stromal cells were sometimes present (Figure S2a). Using LDH leakage as readout, we also observed increased drug-induced cell death in CRC-PDE challenged with 5-FU, compared to the vehicle control (*N* = 5/5) (Figure 4).

Drug-induced cell death ranged from 20 to about 70% of the total CRC-PDE, depending on the case (Figure 4). Differences were also observed for the three CRC-PDE cultures challenged with a combination of 5-FU and Oxaliplatin (OXA, Figure S2b). These data suggest distinct drug sensitivities for CRC-PDE derived from different cases.

## 4. Discussion

CRC heterogeneity and the native TME play important roles in drug sensitivity, thus their preservation is expected to improve representability and predictive value of pre- and co-clinical models [26]. Therefore, patient-derived models are expected to be relevant tools to address clinical translational questions such as tumour and TME heterogeneity, progression, and drug sensitivity. In PDX, tumour heterogeneity can be maintained, but stromal and immune are absent or progressively replaced by host cells [45]. Ex vivo cultures are mainly limited by the short duration and rapid loss of the original cellular phenotypic features. Herein, we demonstrated that CRC-PDE cultures can be generated from fresh surgical resection specimens, without the need for exogenous matrices or passage in immunocompromised mice [46]. CRC-PDE cultures were successfully established from all tumour samples, and 80% of the cultures were maintained for at least 28 days; shorter culture duration was mainly due to extensive sampling. PDE retained a complex 3D organisation lacking in many preclinical models and characteristic of tumour tissues, with partial preservation of tumour cells, stromal matrix, fibroblasts and inflammatory cells. We successfully cultured tumour samples from major different pathways of CRC pathogenesis: MSI-high phenotype; MSS with *BRAF*V600E, possibly representative of a CIMP-positive phenotype; *KRAS* mutated tumours. The non-MSI-high tumours possibly represent cases with CIN, the most common form of genetic instability in CRC, for which there is a lack of alternatives when standard chemotherapy fails. Therefore, the CRC-PDE model may be appropriate to study and target CRC with CIN phenotype, to improve our understanding of the most common form of genetic instability in CRC [47] and to develop new therapeutic options. We took advantage of agitation-based systems to establish a simple methodology culture of CRC-PDE, which does not resource to artificial or animal-derived scaffolds and is easy to sample. Culture under agitation is reported by us and others to present benefits in terms of oxygen and soluble factor diffusion [48,49,50]. Recently, we have successfully applied it for breast and ovarian carcinoma explant culture [33,51]. CRC-PDE size decrease during culture, concomitant with concentration increase, points to the hypothesis of fragmentation of the original PDE into smaller ones along culture time. High cell viability was detected in PDE during culture, despite the decrease in PDE cellularity. This can be associated initially with the shedding of the dead cells derived from the mechanical processing, and later with the senescent and less proliferative phenotype observed for tumour cells and partial loss of other cell populations. Interestingly, over time, tumour cells showed a preferential peripheral location within PDE. This spatial distribution could reflect an adaptation to the culture setting, as the periphery should offer the best exposure to culture medium and oxygen. Nonetheless, we have not observed zonation in ovarian and breast cancer explants [51] cultured under agitation and previous reports state that PDX-derived slice thickness was not a major determinant for cell viability [52]. Curiously, in vitro reports using spheroids of a lung cancer cell line combined with stromal cells also exhibited this spatial organisation [53]. Therefore, other potential factors may include tissue compactness, dependent on extracellular matrix composition, cellularity, stromal composition and density, as well as cell metabolic status. Multiple CRC preclinical models have been developed, namely PDX and in vitro/ex vivo approaches using patient-derived material. PDX can retain original mutational tumour features, but they are labour, cost intensive [54,55,56,57] and can exhibit deregulated genetic pathways, such as upregulation of proliferative genes and downregulation of immune-related ones [58]. The majority of in vitro models rely solely on the presence of tumour epithelial cells or exhibit short-term duration (few days) [28,29,59,60]. In comparison to state-of-the-art methods, such as patient-derived organoids, in PDE we were able to retain both tumour cells and features of TME that cannot be captured in epithelial-restricted organoids [61]. Recently, metastatic CRC patients that received organoid-guided treatment choice did not show clinical benefit [62], highlighting the need for additional studies on their therapeutic predictability. Although limited by initial sample size, CRC-PDE require a small amount of original tumour to achieve multiple explants per sample. These explants are maintained in parallel and can be simultaneously used for different drug studies. However, contrasting to the proliferative nature of organoids, CRC-PDE could not be expanded. Generating organoids from the same CRC samples could tackle the non-proliferative nature of CRC-PDE, despite current limitations on deriving colorectal cancer organoids (culture success rates have been reported from 50 to 70% in CRC) [61]. Other ex vivo models that have been developed for CRC or CRC hepatic metastasis, such as tissue slice cultures, exhibit short culture duration (up to 3 days) and often are poorly characterised [63,64]. We performed a thorough histological characterisation of CRC-PDE along 4 weeks of culture. In cultures established from 23 distinct tumours, key morphologic, immunophenotypic and genetic characteristics of the original tumours were sustained. From our data, CRC-PDE seem genetically stable, as amongst the driver mutations analysed, no additional ones emerged during culture time and the ones observed initially were consistently present afterwards. However, it is important to note that there were changes in specific features, such as cellularity, the proliferative status of the epithelial population and cell ratios of TME populations. One case showed a different major genetic alteration (*KRAS* mutation) since day 0, which was probably due to the heterogeneity of the parental tumour causing sampling bias, although we were unable to trace back the genetic alteration in the patient matched-parental tumour or lymph node metastasis. The presence of *KRAS* mutations could impact the treatment management [65], therefore it could be clinically relevant to confirm this intratumoral heterogeneity. Our results provide insight into the dynamics of tumour subclones over extended culture, as 40% of ten PDE with *KRAS* mutated cells showed a positive selection of these clones over time. This selection could reflect clonal competition within the tumour, possibly reflecting a faster growth as observed by Mousavi et al. on CRC spheroids [66], and suggesting that our model may be suitable for experimental studies including drug predictive assays. Patients for whom chemotherapy is indicated could potentially benefit from chemosensitivity screening in CRC-PDE. As a pilot, we challenged CRC-PDE derived from five patients with the standard of care, 5-FU. The LDH leakage assay revealed distinct drug sensitivities in cultures derived from different patients, that could not be identified by HE. This data corroborates our recent report on the suitability of the LDH assay for evaluation of drug-induced cell death in explant cultures, as it is not dependent upon the integrity of the sample at the experimental endpoint [67]. To address the translation potential of the model, it will be essential to expand the dataset of drug exposure and to collect data from patient follow-up, including the detection and quantification of *KRAS* mutations on tumour recurrences (locally or in metastatic disease), and clinical outcome. Overall, the rapid establishment of CRC-PDE cultures and their long duration are advantages for their application to guide patient treatment selection and potentially avoid overtreatment of non-responsive disease in the adjuvant setting. As long as the temporal dynamics of CRC-PDE cultures are recognised, the CRC-PDE methodology can be a useful tool to address clinical translational questions such as tumour heterogeneity and progression, as well as drug sensitivity.

## 5. Conclusions

We established long-term, scaffold-free cultures of CRC patient-derived explants with detailed histopathological characterisation and preservation of original tumour key genetic features involved in CRC carcinogenesis. This model constitutes a potential preclinical or co-clinical tool to predict therapeutic outcomes. Importantly, recognition of the temporal dynamics may be critical to support the translational value of the patient-derived models.

## Figures and Tables

**Table 1:** Clinicopathological features of the 26 patients.

Clinicopathological Feature	Number of Patients (%)
Sex	
Female	7 (26.9)
Male	19 (73.1)
Age	
Median	68
Range	51–87
Tumour location	
Ascending colon	5 (19.2)
Rectum	1 (3.8)
Sigmoid	18 (69.2)
Transverse colon	2 (7.7)
Tumour histological type	
Adenocarcinoma NOS	20 (76.9)
Adenocarcinoma with mucinous component	4 (15.4)
Mucinous adenocarcinoma	2 (7.7)
Tumour grade (WHO)	
Low	26 (100)
Tumour TNM	
pT1 N0	3 (11.5)
pT2 N0	5 (19.2)
pT3 N0	7 (26.9)
pT3 N1	4 (15.4)
pT3(m) N0	3 (11.5)
pT4a N0	1 (3.8)
pT4a N1	3 (11.5)
Tumour stage	
I	8 (30.8)
IIA	10 (38.5)
IIB	1 (3.8)
IIIB	6 (23.1)
IIIC	1 (3.8)
Follow-up, median in years (range)	2 (0–4.75)
Chemotherapy for CRC	5 (19.2)

CRC, colorectal cancer; NOS, not otherwise specified.

## Data Availability

The data presented in this study are available in the article or supplementary files.

## Supplementary Materials

The following are available online at https://www.mdpi.com/article/10.3390/cancers13184695/s1, Figure S1: Morphological characterisation, Figure S2: Drug challenge in CRC-PDE cultures, Figure S3: Representative electropherogram, Table S1: Scores of histopathological characterisation of 21 CRC-PDEs relative to day 0, Table S2: Clinicopathological features of the 26 patients and respective original tumours.

## References

[1] Labianca R., Nordlinger B., Beretta G.D., Mosconi S., Mandalà M., Cervantes A., Arnold D. (2013). Early Colon Cancer: ESMO Clinical Practice Guidelines for Diagnosis, Treatment and Follow-Up. Ann. Oncol..

[2] Bray F., Ren J.S., Masuyer E., Ferlay J. (2013). Global Estimates of Cancer Prevalence for 27 Sites in the Adult Population in 2008. Int. J. Cancer.

[3] Al-Sohaily S., Biankin A., Leong R., Kohonen-Corish M., Warusavitarne J. (2012). Molecular Pathways in Colorectal Cancer. J. Gastroenterol. Hepatol..

[4] Jover R., Nguyen T., Prezcarbonell L., Zapater P., Pay A., Alenda C., Rojas E., Cubiella J., Balaguer F., Morillas J.D. (2011). 5-Fluorouracil Adjuvant Chemotherapy Does Not Increase Survival in Patients with CpG Island Methylator Phenotype Colorectal Cancer. Gastroenterology.

[5] Van Cutsem E., Cervantes A., Nordlinger B., Arnold D., The ESMO Guidelines Working Group (2014). Metastatic Colorectal Cancer: ESMO Clinical Practice Guidelines for Diagnosis, Treatment and Follow-Up. Ann. Oncol..

[6] Linnekamp J.F., Wang X., Medema J.P., Vermeulen L. (2015). Colorectal Cancer Heterogeneity and Targeted Therapy: A Case for Molecular Disease Subtypes. Cancer Res..

[7] Van Cutsem E., Lenz H.-J., Köhne C.-H., Heinemann V., Tejpar S., Melezínek I., Beier F., Stroh C., Rougier P., van Krieken J.H. (2015). Fluorouracil, Leucovorin, and Irinotecan Plus Ctations in Colorectal Cancer.Etuximab Treatment and RAS Mu. J. Clin. Oncol..

[8] Nyga A., Loizidou M., Emberton M., Cheema U. (2013). A Novel Tissue Engineered Three-Dimensional in Vitro Colorectal Cancer Model. Acta Biomater..

[9] Junttila M.R., de Sauvage F.J. (2013). Influence of Tumour Micro-Environment Heterogeneity on Therapeutic Response. Nature.

[10] Deschoolmeester V., Baay M., Lardon F., Pauwels P., Peeters M. (2011). Immune Cells in Colorectal Cancer: Prognostic Relevance and Role of MSI. Cancer Microenviron..

[11] Erreni M., Mantovani A., Allavena P. (2011). Tumor-Associated Macrophages (TAM) and Inflammation in Colorectal Cancer. Cancer Microenviron..

[12] Frey D.M., Droeser R.A., Viehl C.T., Zlobec I., Lugli A., Zingg U., Oertli D., Kettelhack C., Terracciano L., Tornillo L. (2010). High Frequency of Tumor-Infiltrating FOXP3+ Regulatory T Cells Predicts Improved Survival in Mismatch Repair-Proficient Colorectal Cancer Patients. Int. J. Cancer.

[13] Herrera M., Herrera A., Domínguez G., Silva J., García V., García J.M., Gómez I., Soldevilla B., Muñoz C., Provencio M. (2013). Cancer-Associated Fibroblast and M2 Macrophage Markers Together Predict Outcome in Colorectal Cancer Patients. Cancer Sci..

[14] Lu J., Sceusi E., Zhou Y., Tachibani I., Maru D.M., Hawke D.H. (2014). Endothelial Cells Promote the Colorectal Cancer Stem Cell Phenotype Through a Soluble Form of Jagged-1. Cancer Cell.

[15] Seyhan A.A., Carini C. (2019). Are Innovation and New Technologies in Precision Medicine Paving a New Era in Patients Centric Care?. J. Transl. Med..

[16] Majumder B., Baraneedharan U., Thiyagarajan S., Radhakrishnan P., Narasimhan H., Dhandapani M., Brijwani N., Pinto D.D., Prasath A., Shanthappa B.U. (2015). Predicting Clinical Response to Anticancer Drugs Using an Ex Vivo Platform That Captures Tumour Heterogeneity. Nat. Commun..

[17] Asghar W., El Assal R., Shafiee H., Pitteri S., Paulmurugan R., Demirci U. (2015). Engineering Cancer Microenvironments for in Vitro 3-D Tumor Models. Mater. Today.

[18] Luca A.C., Mersch S., Deenen R., Schmidt S., Messner I., Schäfer K.L., Baldus S.E., Huckenbeck W., Piekorz R.P., Knoefel W.T. (2013). Impact of the 3D Microenvironment on Phenotype, Gene Expression, and EGFR Inhibition of Colorectal Cancer Cell Lines. PLoS ONE.

[19] Golovko D., Kedrin D., Yilmaz Ö.H., Roper J. (2015). Colorectal Cancer Models for Novel Drug Discovery. Expert Opin. Drug Discov..

[20] Morgan M.M., Johnson B.P., Livingston M.K., Schuler L.A., Alarid E.T., Sung K.E., Beebe D.J. (2016). Personalized in Vitro Cancer Models to Predict Therapeutic Response: Challenges and a Framework for Improvement. Pharmacol. Ther..

[21] Neal J.T., Kuo C.J. (2016). Organoids as Models for Neoplastic Transformation. Annu. Rev. Pathol. Mech. Dis..

[22] Van De Wetering M., Francies H.E., Francis J.M., Bounova G., Iorio F., Pronk A., Van Houdt W., Van Gorp J., Taylor-Weiner A., Kester L. (2015). Prospective Derivation of a Living Organoid Biobank of Colorectal Cancer Patients. Cell.

[23] Bleijs M., Wetering M., Clevers H., Drost J. (2019). Xenograft and Organoid Model Systems in Cancer Research. EMBO J..

[24] Ooft S.N., Weeber F., Dijkstra K.K., McLean C.M., Kaing S., van Werkhoven E., Schipper L., Hoes L., Vis D.J., van de Haar J. (2019). Patient-Derived Organoids Can Predict Response to Chemotherapy in Metastatic Colorectal Cancer Patients. Sci. Transl. Med..

[25] Weeber F., Van De Wetering M., Hoogstraat M., Dijkstra K.K., Krijgsman O., Kuilman T., Gadellaa-Van Hooijdonk C.G.M., Van Der Velden D.L., Peeper D.S., Cuppen E.P.J.G. (2015). Preserved Genetic Diversity in Organoids Cultured from Biopsies of Human Colorectal Cancer Metastases. Proc. Natl. Acad. Sci. USA.

[26] Powley I.R., Patel M., Miles G., Pringle H., Howells L., Thomas A., Kettleborough C., Bryans J., Hammonds T., MacFarlane M. (2020). Patient-Derived Explants (PDEs) as a Powerful Preclinical Platform for Anti-Cancer Drug and Biomarker Discovery. Br. J. Cancer.

[27] Baker L.A., Tiriac H., Clevers H., Tuveson D.A. (2016). Modeling Pancreatic Cancer with Organoids. Trends Cancer.

[28] Ashley N., Jones M., Ouaret D., Wilding J., Bodmer W.F. (2014). Rapidly Derived Colorectal Cancer Cultures Recapitulate Parental Cancer Characteristics and Enable Personalized Therapeutic Assays. J. Pathol..

[29] Jeppesen M., Hagel G., Glenthoj A., Vainer B., Ibsen P., Harling H., Thastrup O., Jørgensen L.N., Thastrup J., Jorgensen L.N. (2017). Short-Term Spheroid Culture of Primary Colorectal Cancer Cells as an in Vitro Model for Personalizing Cancer Medicine. PLoS ONE.

[30] Ahmed M., Jinks N., Babaei-Jadidi R., Kashfi H., Castellanosuribe M., May S.T., Mukherjee A., Nateri A.S. (2020). Repurposing Antibacterial AM404 as a Potential Anticancer Drug for Targeting Colorectal Cancer Stem-like Cells. Cancers.

[31] Karekla E., Liao W.J., Sharp B., Pugh J., Reid H., Le Quesne J., Moore D., Pritchard C., MacFarlane M., Pringle J.H. (2017). Ex Vivo Explant Cultures of Non-Small Cell Lung Carcinoma Enable Evaluation of Primary Tumor Responses to Anticancer Therapy. Cancer Res..

[32] Tognon C.E., Sears R.C., Mills G.B., Gray J.W., Tyner J.W. (2020). Ex Vivo Analysis of Primary Tumor Specimens for Evaluation of Cancer Therapeutics. Annu. Rev. Cancer Biol..

[33] Abreu S., Silva F., Mendes R., Mendes T.F., Teixeira M., Santo V.E., Boghaert E.R., Félix A., Brito C. (2020). Patient-Derived Ovarian Cancer Explants: Preserved Viability and Histopathological Features in Long-Term Agitation-Based Cultures. Sci. Rep..

[34] Sato T., Vries R.G., Snippert H.J., van de Wetering M., Barker N., Stange D.E., van Es J.H., Abo A., Kujala P., Peters P.J. (2009). Single Lgr5 Stem Cells Build Crypt-Villus Structures in Vitro without a Mesenchymal Niche. Nature.

[35] Fujii M., Shimokawa M., Date S., Takano A., Matano M., Nanki K., Ohta Y., Toshimitsu K., Nakazato Y., Kawasaki K. (2016). A Colorectal Tumor Organoid Library Demonstrates Progressive Loss of Niche Factor Requirements during Tumorigenesis. Cell Stem Cell.

[36] Schneider C.A., Rasband W.S., Eliceiri K.W. (2012). NIH Image to ImageJ: 25 Years of Image Analysis. Nat. Methods.

[37] Schindelin J., Arganda-Carreras I., Frise E., Kaynig V., Longair M., Pietzsch T., Preibisch S., Rueden C., Saalfeld S., Schmid B. (2012). Fiji: An Open-Source Platform for Biological-Image Analysis. Nat. Methods.

[38] Herranz N., Gil J. (2018). Mechanisms and Functions of Cellular Senescence. J. Clin. Investig..

[39] Cho K.A., Sung J.R., Yoon S.O., Ji H.P., Jung W.L., Kim H.P., Kyung T.K., Ik S.J., Sang C.P. (2004). Morphological Adjustment of Senescent Cells by Modulating Caveolin-1 Status. J. Biol. Chem..

[40] Prall F., Hühns M. (2019). Quantitative Evaluation of TP53 Immunohistochemistry to Predict Gene Mutations: Lessons Learnt from a Series of Colorectal Carcinomas. Hum. Pathol..

[41] Rodriguez-Bigas M.A., Boland C.R., Hamilton S.R., Henson D.E., Jass J.R., Khan P.M., Lynch H., Perucho M., Smyrk T., Sobin L. (1997). A National Cancer Institute Workshop on Hereditary Nonpolyposis Colorectal Cancer Syndrome: Meeting Highlights and Bethesda Guidelines. J. Natl. Cancer Inst..

[42] Umar A., Boland C.R., Terdiman J.P., Syngal S., de la Chapelle A., Rüschoff J., Fishel R., Lindor N.M., Burgart L.J., Hamelin R. (2004). Revised Bethesda Guidelines for Hereditary Nonpolyposis Colorectal Cancer (Lynch Syndrome) and Microsatellite Instability. J. Natl. Cancer Inst..

[43] Silva P., Albuquerque C., Lage P., Fontes V., Fonseca R., Vitoriano I., Filipe B., Rodrigues P., Moita S., Ferreira S. (2016). Serrated Polyposis Associated with a Family History of Colorectal Cancer and/or Polyps: The Preferential Location of Polyps in the Colon and Rectum Defines Two Molecular Entities. Int. J. Mol. Med..

[44] Hoffmann O.I., Ilmberger C., Magosch S., Joka M., Jauch K.W., Mayer B. (2015). Impact of the Spheroid Model Complexity on Drug Response. J. Biotechnol..

[45] Cassidy J.W., Caldas C., Bruna A. (2015). Maintaining Tumor Heterogeneity in Patient-Derived Tumor Xenografts. Cancer Res..

[46] Cai H., Scott E., Kholghi A., Andreadi C., Rufini A., Karmokar A., Britton R.G., Horner-Glister E., Greaves P., Jawad D. (2015). Cancer Chemoprevention: Evidence of a Nonlinear Dose Response for the Protective Effects of Resveratrol in Humans and Mice. Sci. Transl. Med..

[47] Fearon E.R. (2011). Molecular Genetics of Colorectal Cancer. Annu. Rev. Pathol..

[48] Estrada M.F., Rebelo S.P., Davies E.J., Pinto M.T., Pereira H., Santo V.E., Smalley M.J., Barry S.T., Gualda E.J., Alves P.M. (2016). Modelling the Tumour Microenvironment in Long-Term Microencapsulated 3D Co-Cultures Recapitulates Phenotypic Features of Disease Progression. Biomaterials.

[49] Santo V.E., Estrada M.F., Rebelo S.P., Abreu S., Silva I., Pinto C., Veloso S.C., Serra A.T., Boghaert E., Alves P.M. (2016). Adaptable Stirred-Tank Culture Strategies for Large Scale Production of Multicellular Spheroid-Based Tumor Cell Models. J. Biotechnol..

[50] Naipal K.A.T., Verkaik N.S., Sánchez H., van Deurzen C.H.M., den Bakker M.A., Hoeijmakers J.H.J., Kanaar R., Vreeswijk M.P.G., Jager A., van Gent D.C. (2016). Tumor Slice Culture System to Assess Drug Response of Primary Breast Cancer. BMC Cancer.

[51] Cartaxo A.L., Estrada M.F., Domenici G., Roque R., Silva F., Gualda E.J., Loza-Alvarez P., Sflomos G., Brisken C., Alves P.M. (2020). A Novel Culture Method That Sustains ERα Signaling in Human Breast Cancer Tissue Microstructures. J. Exp. Clin. Cancer Res..

[52] Davies E.J., Dong M., Gutekunst M., Närhi K., van Zoggel H.J.A.A.A.A., Blom S., Nagaraj A., Metsalu T., Oswald E., Erkens-Schulze S. (2015). Capturing Complex Tumour Biology in Vitro: Histological and Molecular Characterisation of Precision Cut Slices. Sci. Rep..

[53] Lamichhane S.P., Arya N., Kohler E., Xiang S., Christensen J., Shastri V.P. (2016). Recapitulating Epithelial Tumor Microenvironment in Vitro Using Three Dimensional Tri-Culture of Human Epithelial, Endothelial, and Mesenchymal Cells. BMC Cancer.

[54] Gao H., Korn J.M., Ferretti S., Monahan J.E., Wang Y., Singh M., Zhang C., Schnell C., Yang G., Zhang Y. (2015). High-Throughput Screening Using Patient-Derived Tumor Xenografts to Predict Clinical Trial Drug Response. Nat. Med..

[55] Lazzari L., Corti G., Picco G., Isella C., Montone M., Arcela P., Durinikova E., Zanella E.R., Novara L., Barbosa F. (2019). Patient-Derived Xenografts and Matched Cell Lines Identify Pharmacogenomic Vulnerabilities in Colorectal Cancer. Clin. Cancer Res..

[56] Julien S., Merino-Trigo A., Lacroix L., Pocard M., Goeŕé D., Mariani P., Landron S., Bigot L., Nemati F., Dartigues P. (2012). Characterization of a Large Panel of Patient-Derived Tumor Xenografts Representing the Clinical Heterogeneity of Human Colorectal Cancer. Clin. Cancer Res..

[57] Uronis J.M., Osada T., McCall S., Yang X.Y., Mantyh C., Morse M.A., Lyerly H.K., Clary B.M., Hsu D.S. (2012). Histological and Molecular Evaluation of Patient-Derived Colorectal Cancer Explants. PLoS ONE.

[58] Monsma D.J., Monks N.R., Cherba D.M., Dylewski D., Eugster E., Jahn H., Srikanth S., Scott S.B., Richardson P.J., Everts R.E. (2012). Genomic Characterization of Explant Tumorgraft Models Derived from Fresh Patient Tumor Tissue. J. Transl. Med..

[59] Lee S.H., Hong J.H., Park H.K., Park J.S., Kim B.K., Lee J.Y., Jeong J.Y., Yoon G.S., Inoue M., Choi G.S. (2015). Colorectal Cancer-Derived Tumor Spheroids Retain the Characteristics of Original Tumors. Cancer Lett..

[60] Forsythe S., Mehta N., Devarasetty M., Sivakumar H., Gmeiner W., Soker S., Votanopoulos K., Skardal A. (2020). Development of a Colorectal Cancer 3D Micro-Tumor Construct Platform From Cell Lines and Patient Tumor Biospecimens for Standard-of-Care and Experimental Drug Screening. Ann. Biomed. Eng..

[61] Drost J., Clevers H. (2018). Organoids in Cancer Research. Nat. Rev. Cancer.

[62] Ooft S.N., Weeber F., Schipper L., Dijkstra K.K., McLean C.M., Kaing S., van de Haar J., Prevoo W., van Werkhoven E., Snaebjornsson P. (2021). Prospective Experimental Treatment of Colorectal Cancer Patients Based on Organoid Drug Responses. ESMO Open.

[63] Martin S.Z., Wagner D.C., Hörner N., Horst D., Lang H., Tagscherer K.E., Roth W. (2019). Ex Vivo Tissue Slice Culture System to Measure Drug-Response Rates of Hepatic Metastatic Colorectal Cancer. BMC Cancer.

[64] Sönnichsen R., Hennig L., Blaschke V., Winter K., Körfer J., Hähnel S., Monecke A., Wittekind C., Jansen-Winkeln B., Thieme R. (2018). Individual Susceptibility Analysis Using Patient-Derived Slice Cultures of Colorectal Carcinoma. Clin. Colorectal Cancer.

[65] Misale S., Yaeger R., Hobor S., Scala E., Janakiraman M., Liska D., Valtorta E., Schiavo R., Buscarino M., Siravegna G. (2012). Emergence of KRAS Mutations and Acquired Resistance to Anti-EGFR Therapy in Colorectal Cancer. Nature.

[66] Mousavi N., Truelsen S.L.B., Hagel G., Jorgensen L.N., Harling H., Timmermans V., Melchior L.C., Thysen A.H., Heegaard S., Thastrup J. (2019). KRAS Mutations in the Parental Tumour Accelerate in Vitro Growth of Tumoroids Established from Colorectal Adenocarcinoma. Int. J. Exp. Pathol..

[67] Cox M.C., Mendes R., Silva F., Mendes T.F., Zelaya-Lazo A., Halwachs K., Purkal J.J., Isidro I.A., Felix A., Boghaert E.R. (2021). Application of LDH assay for therapeutic efficacy evaluation of ex vivo tumor models. Sci. Rep..

