# Supplementary material for: Patient-Derived Explants of Colorectal Cancer: Histopathological and Molecular Analysis of Long-Term Cultures
Source: Cancers (Basel). 2021 Sep 19;13(18):4695. doi: 10.3390/cancers13184695 (PMC8465429; doi:10.3390/cancers13184695)
Supplement: Supplementary file 1 [file cancers-13-04695-s001.zip › cancers-1383702-supplementary.pdf]

Article – Supplementary Material

Patient-Derived Explants of Colorectal Cancer: Histopathological and Molecular Analysis of Long-Term Cultures

Sara da Mata[1,2,†] & Teresa Franchi-Mendes [3,4,†], Sofia Abreu [3,4], Bruno Filipe [5], Sónia Morgado [1], Marta Mesquita [1], Vítor E. Santo [3,4], Cristina Albuquerque [5], Ricardo Fonseca [1,6], Erwin Boghaert [7], Isadora Rosa [8,*], Catarina Brito [3,4,9,*]

[1] Serviço de Anatomia Patológica, Instituto Português de Oncologia de Lisboa Francisco Gentil (IPOLFG, EPE), R. Prof. Lima Basto, 1099-023 Lisboa, Portugal. smata@ipolisboa.min-saude.pt (S.d.M.); smorgado@ipolisboa.min-saude.pt (S.M.); mmesquita@ipolisboa.min-saude.pt (M.M.); rifonseca@ipolisboa.min-saude.pt (R.F.)
[2] NOVA Medical School, NMS, Universidade Nova de Lisboa, Campo dos Mártires da Pátria 130, 1169-056 Lisboa, Portugal
[3] IBET Instituto de Biologia Experimental e Tecnológica, Apartado 12, 2780-901 Oeiras, Portugal. mtmendes@ibet.pt (T.F.M.); sofia_abreu5@hotmail.com (S.A.); vitorespsanto@gmail.com (V.E.S.); anabrito@ibet.pt (C.B.)
[4] Instituto de Tecnologia Química e Biológica António Xavier, Universidade Nova de Lisboa, Av. da República, 2780-157 Oeiras, Portugal
[5] Unidade de Investigação em Patobiologia Molecular (UIPM), Instituto Português de Oncologia de Lisboa Francisco Gentil (IPOLFG, EPE), R. Prof. Lima Basto, 1099-023 Lisboa, Portugal. bfil-ipe@ipolisboa.min-saude.pt (B.F.); calbuque@ipolisboa.min-saude.pt (C.A.)
[6] FMUL, Faculdade de Medicina da Universidade de Lisboa, Av. Prof. Egas Moniz MB, 1649-028 Lis-boa, Portugal
[7] Abbvie, 1 North Waukegan Road, North Chicago, IL 60064-6098, United States of America; er-win.boghaert@abbvie.com
[8] Serviço de Gastrenterologia, Instituto Português de Oncologia de Lisboa Francisco Gentil (IPOLFG, EPE), R. Prof. Lima Basto, 1099-023 Lisboa, Portugal. isarosa@ipolisboa.min-saude.pt (I.R.)
[9] The Discoveries Centre for Regenerative and Precision Medicine, Lisbon Campus, Av. da República, 2780-157 Oeiras Portugal
† Sara da Mata and Teresa F Mendes equally contributed to this work.
* Correspondence: I.R.: isarosa@ipolisboa.min-saude.pt; C.B.: anabrito@ibet.pt

Figure S1

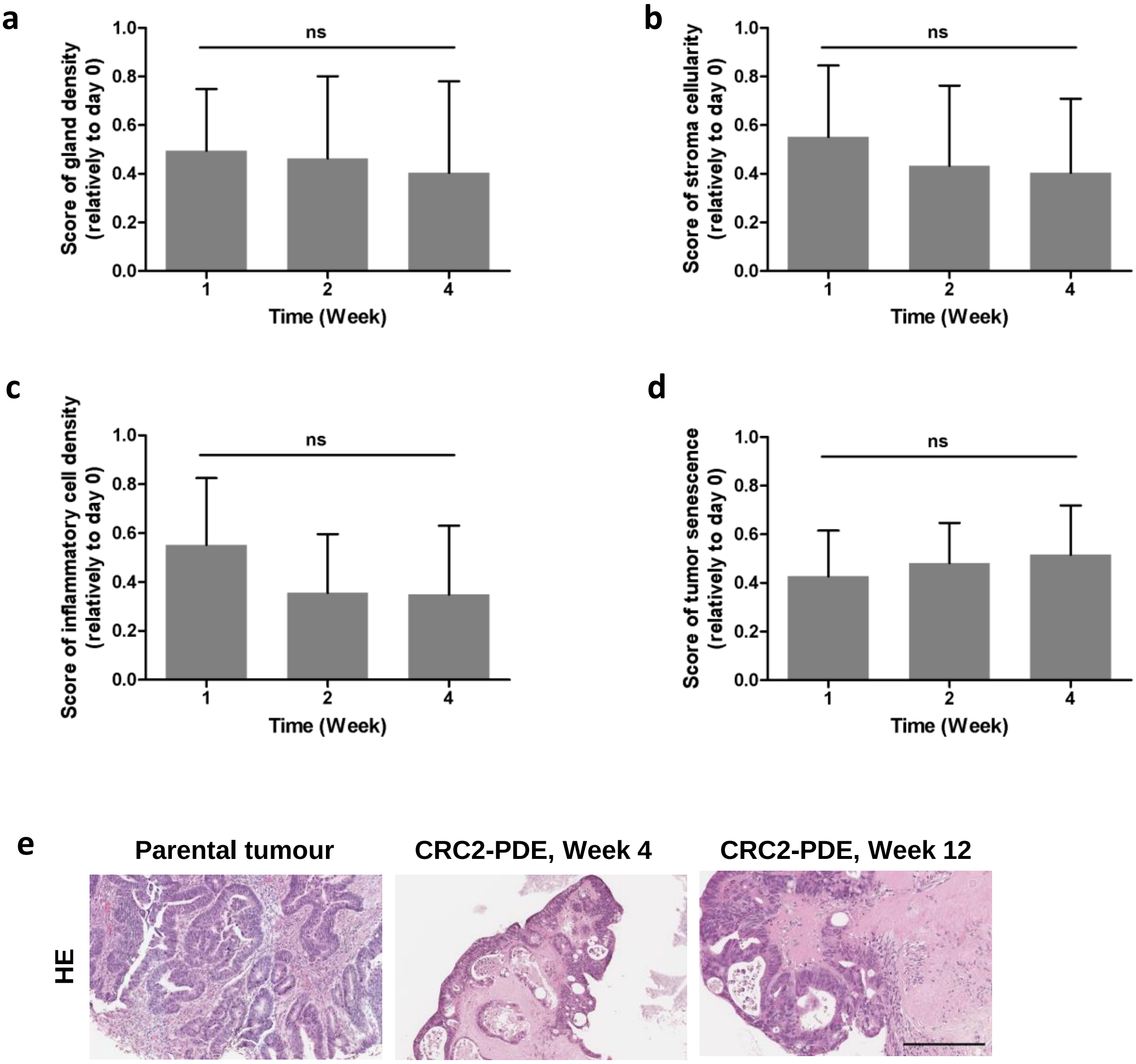

Supplementary Figure S1: Morphological characterization of CRC-PDE. Scores of (**a**) gland density, (**b**) stroma cellularity, (**c**) inflammatory cells and (**d**) tumour cell senescence were evaluated along 4 weeks of culture from 21 biological replicates. Data are presented as mean ± SD (score for each case is described in Figure 2e and Table S1). Data were analysed by one-way Friedman's test. Week 1: days 7-9; Week 2: days 14-17; Week 4: days 25-28. (**e**) Representative imagens of morphological characterization by HE of the parental tumour (CRC2) and derived explants (CRC2-PDE), at weeks 4 and 12 of culture. Tumour cells formed glands and located preferentially in the periphery. Tumoral necrosis was focally present. Scale bar 200 μm. *CRC-PDE,* Colorectal cancer patient-derived explants; *HE,* hematoxylin and eosin,. *ns*, non-significant.

Figure S2

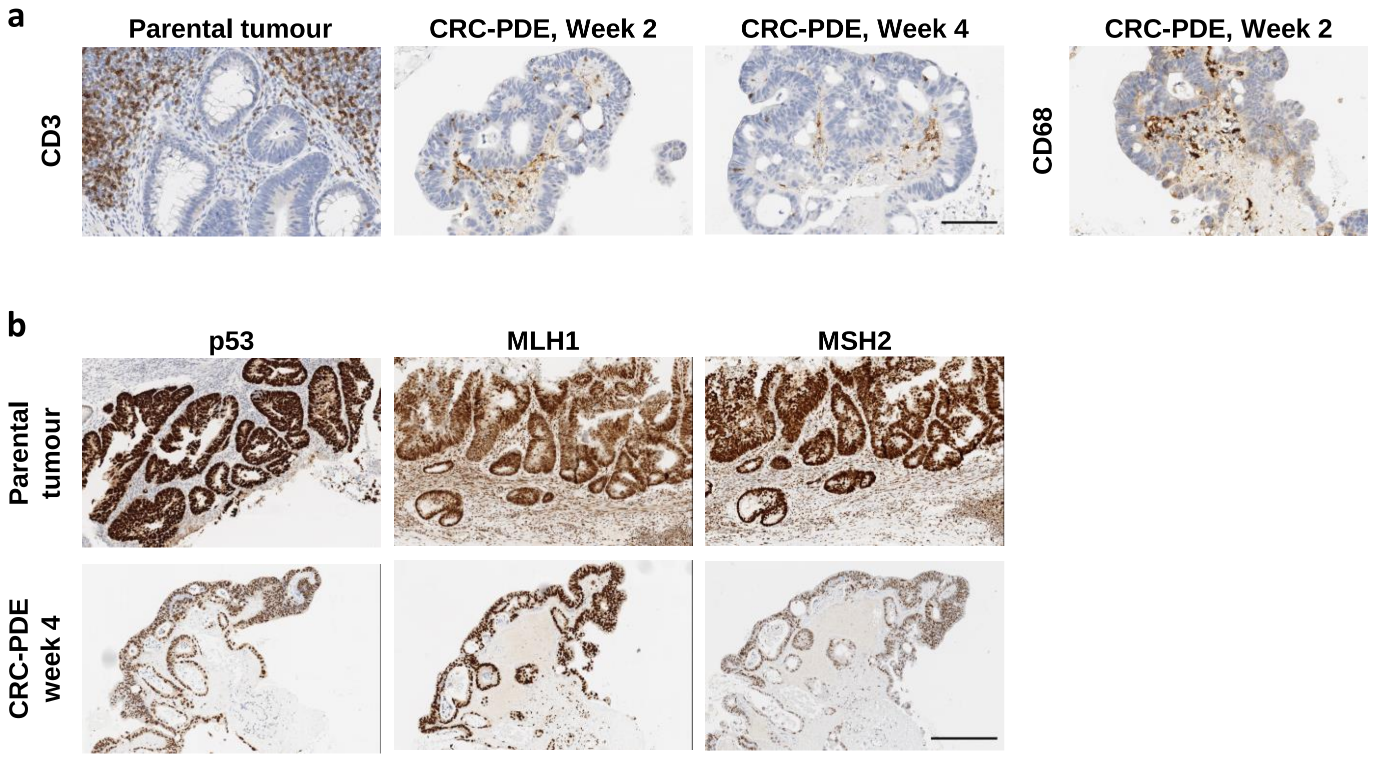

Supplementary Figure S2: Immunohistochemical characterization of CRC-PDE. (**a**) Representative images of detection of T lymphocytes (CD3-positive) and macrophages (CD68-positive) on the parental tumour tissue (CRC2) and derived explants (CRC2-PDE), at weeks 2 and 4 of culture. Scale bar 100 μm. (**b**) Representative images of detection of proteins associated with carcinogenesis pathways in the parental tumour (CRC2) and derived explants (CRC2-PDE), at week 4 of culture. Scale bar 300 μm. *CRC-PDE,* Colorectal cancer patient-derived explants; *MLH1,* human mutL homolog 1; *MSH2,* human mutS homolog 2.

Figure S3

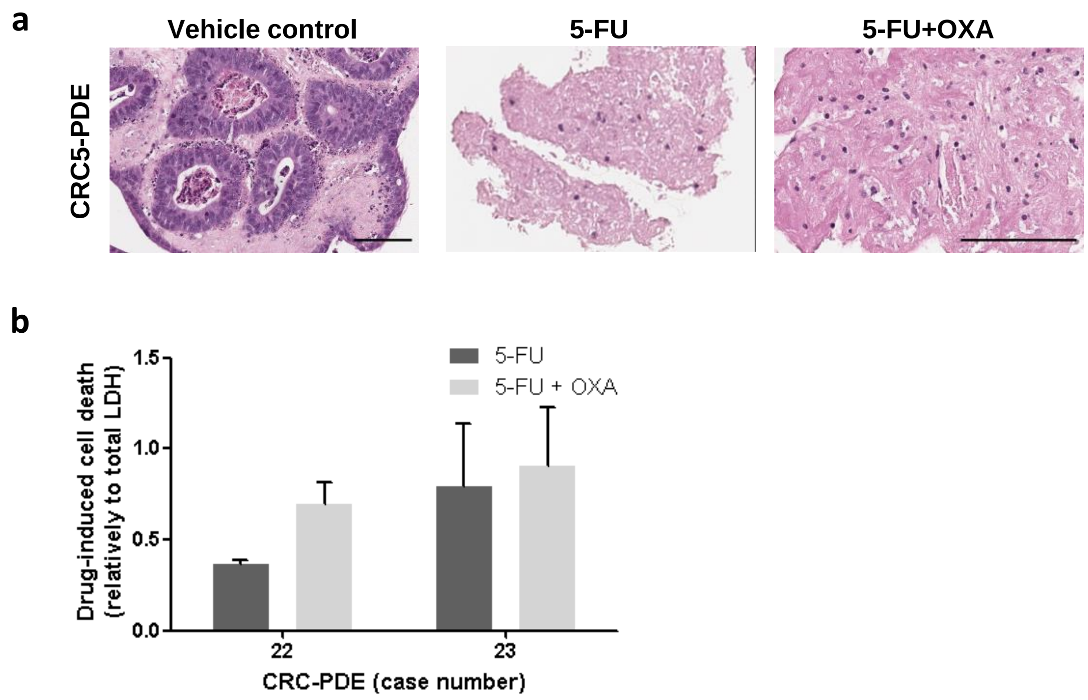

Supplementary Figure S3: Drug challenge in CRC-PDE cultures. (**a**) Representative HE images of CRC5-PDE, vehicle control and challenge with 5-FU or5-FU+OXA, at week 3 of culture. After two weeks of drug exposure, control CRC5-PDE had viable tumour while samples treated with 5-FU and 5-FU+OXA showed tumour necrosis and only the latter showed viable stroma. Scale bar 100 µm. **B**, LDH leakage assay of CRC22-PDE and CRC23-PDE exposed to 5-FU and to 5-FU + OXA for 2 weeks relatively to total LDH content. *CRC-PDE,* Colorectal cancer patient-derived explant culture; *5-FU,* 5-Fluorouracil; *HE,* Hematoxylin and eosin; *OXA,* Oxaliplatin.

Figure S4

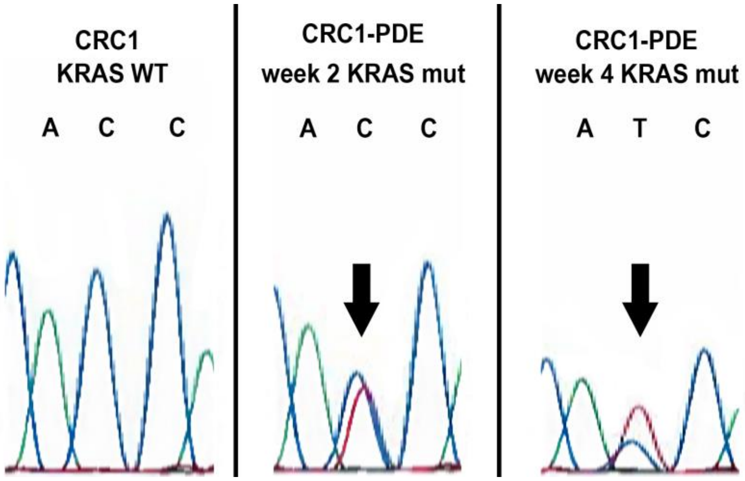

Supplementary Figure S4. CRC1 and CRC1-PDE DNA sequence of *KRAS* exon 2 codon 12. The electropherogram shows the reverse sequence. Arrows indicate *KRAS* mutation. *KRAS* mutated clone enrichment was observed at week 4. *CRC,* Colorectal cancer, *CRC-PDE;* Colorectal cancer patient-derived explants; *WT,* wild type; *mut* mutated.

Table S1. Scores of histopathological characterization of 21 CRC-PDE relative to day 0.

		Time (Day)						
		0	1	4	7-9	11	14-17	25-28
CRC1	**Gland density**	1.0	nd	nd	nd	1.0	0.0	0.0
CRC2		1.0	nd	nd	0.3	nd	0.7	0.7
CRC3		1.0	nd	nd	0.0	nd	0.0	0.0
CRC4		1.0	nd	nd	nd	0.7	0.7	1.0
CRC5		1.0	nd	nd	0.3	nd	0.3	0.0
CRC6		1.0	nd	nd	0.7	nd	0.3	0.3
CRC7		1.0	nd	nd	0.3	nd	0.7	0.7
CRC8		1.0	nd	nd	0.7	nd	0.7	0.7
CRC9		1.0	nd	nd	0.0	nd	0.3	0.3
CRC10		1.0	nd	nd	0.7	nd	0.0	0.0
CRC11		1.0	nd	nd	0.3	nd	0.3	0.3
CRC12		1.0	nd	nd	0.7	nd	0.3	0.3
CRC13		1.0	nd	nd	1.0	nd	1.3	1.3
CRC14		1.0	1.0	0.3	0.3	nd	nd	nd
CRC15		1.0	1.0	0.7	0.7	nd	nd	0.3
CRC16		1.0	0.7	0.3	0.7	nd	nd	nd
CRC17		1.0	nd	nd	nd	nd	nd	0.0
CRC18		1.0	nd	0.7	0.3	nd	nd	0.0
CRC19		1.0	nd	1.0	0.7	nd	0.7	0.7
CRC20		1.0	nd	0.7	0.7	nd	nd	0.7
CRC21		1.0	nd	0.7	0.7	nd	0.7	nd
Mean		1.0	0.9	0.6	0.5	0.8	0.5	0.4
SD		0.0	0.2	0.2	0.3	0.2	0.3	0.4
CRC1	**Stroma cellularity**	1.0	nd	nd	nd	1.0	1.0	0.0
CRC2		1.0	nd	nd	0.7	nd	0.7	0.3
CRC3		1.0	nd	nd	0.0	nd	0.0	0.0
CRC4		1.0	nd	nd	nd	0.3	nd	0.0
CRC5		1.0	nd	nd	0.7	nd	0.3	0.3
CRC6		1.0	nd	nd	0.3	nd	0.0	0.3
CRC7		1.0	nd	nd	0.3	nd	0.3	0.3
CRC8		1.0	nd	nd	0.3	nd	0.0	0.0
CRC9		1.0	nd	nd	1.0	nd	1.0	1.0
CRC10		1.0	nd	nd	0.7	nd	0.3	0.3
CRC11		1.0	nd	nd	0.3	nd	0.3	0.3
CRC12		1.0	nd	nd	0.7	nd	0.7	0.7
CRC13		1.0	nd	nd	0.3	nd	0.7	0.7
CRC14		1.0	1.0	0.3	0.0	nd	nd	nd
CRC15		1.0	1.0	0.7	0.7	nd	nd	0.3
CRC16		1.0	1.0	1.0	1.0	nd	nd	nd
CRC17		1.0	nd	nd	nd	nd	nd	0.7
CRC18		1.0	nd	0.7	0.7	nd	nd	0.3
CRC19		1.0	nd	1.0	1.0	nd	nd	0.7
CRC20		1.0	nd	0.7	0.7	nd	nd	1.0
CRC21		1.0	nd	0.7	0.7	nd	0.3	nd
Mean		1.0	1.0	0.7	0.6	0.7	0.4	0.4
SD		0.0	0.0	0.2	0.3	0.3	0.3	0.3

		Time (Day)						
		0	1	4	7-9	11	14-17	25-28
CRC1	**Senescent phenotype**	0.0	nd	nd	nd	0.3	0.3	0.3
CRC2		0.0	nd	nd	0.0	nd	0.3	0.3
CRC3		0.0	nd	nd	nd	nd	nd	nd
CRC4		0.0	nd	nd	nd	0.7	0.3	0.3
CRC5		0.0	nd	nd	0.3	nd	0.7	0.3
CRC6		0.0	nd	nd	0.3	nd	0.3	0.3
CRC7		0.0	nd	nd	0.3	nd	0.7	1.0
CRC8		0.0	nd	nd	0.3	nd	0.3	0.3
CRC9		0.0	nd	nd	0.3	nd	0.7	nd
CRC10		0.0	nd	nd	0.3	nd	0.7	0.7
CRC11		0.0	nd	nd	0.3	nd	0.7	0.7
CRC12		0.0	nd	nd	0.7	nd	0.7	0.7
CRC13		0.0	nd	nd	0.7	nd	0.3	0.3
CRC14		0.0	0.3	0.7	0.3	nd	nd	nd
CRC15		0.0	0.3	0.7	0.7	nd	nd	0.7
CRC16		0.0	0.3	0.7	0.7	nd	nd	nd
CRC17		0.0	nd	nd	nd	nd	nd	0.3
CRC18		0.0	nd	0.3	0.7	nd	nd	0.7
CRC19		0.0	nd	0.3	0.3	nd	nd	0.7
CRC20		0.0	nd	0.3	0.7	nd	nd	0.7
CRC21		0.0	nd	0.3	0.3	nd	0.3	nd
Mean		0.0	0.3	0.5	0.4	0.5	0.5	0.5
SD		0.0	0.0	0.2	0.2	0.2	0.2	0.2
CRC1	**Inflammatory cell density**	1.0	nd	nd	nd	0.7	0.0	0.0
CRC2		1.0	nd	nd	0.3	nd	0.3	0.3
CRC3		1.0	nd	nd	0.3	nd	0.3	0.0
CRC4		1.0	nd	nd	nd	0.3	nd	0.0
CRC5		1.0	nd	nd	0.7	nd	0.3	0.0
CRC6		1.0	nd	nd	0.3	nd	0.3	0.3
CRC7		1.0	nd	nd	0.7	nd	0.3	0.3
CRC8		1.0	nd	nd	0.3	nd	0.0	0.3
CRC9		1.0	nd	nd	1.0	nd	0.0	1.0
CRC10		1.0	nd	nd	0.7	nd	0.7	0.7
CRC11		1.0	nd	nd	0.7	nd	0.7	0.7
CRC12		1.0	nd	nd	1.0	nd	0.7	0.7
CRC13		1.0	nd	nd	1.0	nd	0.7	0.3
CRC14		1.0	0.3	1.0	0.3	nd	nd	nd
CRC15		1.0	0.7	0.3	0.3	nd	nd	0.0
CRC16		1.0	0.7	0.7	0.3	nd	nd	nd
CRC17		1.0	nd	nd	nd	nd	nd	0.3
CRC18		1.0	nd	1.0	0.3	nd	nd	0.3
CRC19		1.0	nd	1.0	1.0	nd	nd	0.7
CRC20		1.0	nd	0.7	0.3	nd	nd	0.3
CRC21		1.0	nd	0.3	0.3	nd	0.3	nd
Mean		1.0	0.6	0.7	0.6	0.5	0.4	0.3
SD		0.0	0.2	0.3	0.3	0.2	0.2	0.3

Day 0 was considered the reference. It was scored as 1 for gland density, stroma cellularity and inflammatory cell density and as 0 for senescence evaluations.
CRC-PDE, Colorectal cancer patient-derived explants; *nd,* not determined; *SD,* Standard deviation.

Table S2 Clinicopathological features of the 26 patients and respective tumours.

Case	Sex	Age, y	Tumor location	Tumor histological type*	TNM	Stage	*KRAS exon 2*	*BRAF V600E*	MSI	MMRp	p53	Chemo.	FU, in mo	Patient status
CRC1	M	79	Rectum	ADC NOS	pT3 N1b	IIIB	c.35G>A, p.Gly12Asp	WT	MSS	Retained	WT/(+)	No	57	Alive, CRC-F
CRC2	M	54	Sigmoid	ADC NOS	pT1 N0	I	WT	WT	MSS	Retained	(+)	No	33	Dead, UC
CRC3	M	52	Sigmoid	ADC NOS	pT1 N0	I	WT	WT	MSI-L	Retained	WT	No	51	Alive, CRC-F
CRC4	M	66	Sigmoid	ADC NOS	pT3m N0	IIA	WT	WT	MSS	Retained	(+)	No	43	Alive, CRC-F
CRC5	M	72	Sigmoid	ADC NOS	pT4a N0	IIB	WT	WT	MSS	Retained	Null	No	0	Dead, PO
CRC6	F	74	Sigmoid	ADC NOS	pT3 N1a	IIIB	c.34G>T, p.Gly12Asp	WT	MSS	Retained	Null	Yes	11	Alive, CRC-F
CRC7	M	68	Sigmoid	ADC NOS	pT3 N0	IIA	c.35G>T, p.Gly12Val	WT	MSS	Retained	Null	No	46	Alive, CRC-F
CRC8	M	87	Sigmoid	ADC NOS	pT2 N0	I	c.35G>A, p.Gly12Asp	WT	MSS	Retained	Null	No	2	Alive, CRC-F
CRC9	M	77	Sigmoid	ADC NOS	pT3 N0	IIA	WT	WT	MSI-L	Retained	(+)	No	0	Dead, PO
CRC10	F	64	Ascending colon	ADC NOS	pT3 N0	IIA	WT	Mutated	MSS	Retained	WT	No	36	Alive, CRC-F
CRC11	F	51	Ascending colon	ADC with mucinous component	(m)pT3 N0	IIA	c.38G>A, p.Gly13Asp	WT	MSI-H	PMS2 loss	WT	No	8	Dead, OC
CRC12	M	63	Sigmoid	Mucinous ADC	pT3 N0	IIA	c.34G>T, p.Gly12Cys	WT	MSS	Retained	(+)	No	45	Alive, CRC-F
CRC13	M	65	Ascending colon	ADC NOS	pT2 N0	I	WT	WT	MSS	Retained	Null	No	8	Dead, OC
CRC14	M	80	Sigmoid	ADC NOS	(m)pT3 N0	IIA	WT	WT	MSS	Retained	Null	No	0	Dead, PO
CRC15	M	59	Transverse colon	ADC NOS	pT4a N1b	IIIB	c.34G>A, p.Gly12Ser	WT	MSS	Retained	Null	Yes	39	Alive, CRC
CRC16	F	78	Ascending colon	ADC with mucinous component	pT3 N1b	IIIB	c.38G>A, p.Gly13Asp	NA	NA	Retained	(+)	Yes	36	Alive, CRC
CRC17	M	60	Sigmoid	Mucinous ADC	pT4b N1c	IIIC	c.35G>T, p.Gly12Val	NA	NA	Retained	Null	Yes	11	Alive, CRC
CRC18	F	72	Ascending colon	ADC with mucinous component	pT2 N0	I	c.35G>T, p.Gly12Val	NA	NA	Retained	WT	No	23	Alive, CRC-F
CRC19	M	72	Sigmoid	ADC NOS	pT2 N0	I	NA	NA	NA	Retained	(+)	No	31	Alive, CRC-F
CRC20	M	60	Sigmoid	ADC NOS	pT4a N1b	IIIB	NA	NA	NA	Retained	(+)	Yes	4	Dead, CRC
CRC21	F	75	Sigmoid	ADC NOS	pT3 N0	IIA	WT	NA	NA	Retained	Null	No	30	Alive, CRC-F
CRC22	M	74	Sigmoid	ADC NOS	pT3 N0	II	NA	NA	NA	NA	NA	No	31	Alive, CRC-F
CRC23	M	69	Sigmoid	ADC NOS	pT1 N0	I	NA	NA	NA	NA	NA	No	5	Dead, OC
CRC24	M	68	Sigmoid	ADC NOS	pT3 N0	II	NA	NA	NA	Retained	NA	No	13	Dead, OC
CRC25	F	60	Sigmoid	ADC NOS	pT3 N1	IIIB	NA	NA	NA	Retained	NA	No	22	Alive, CRC-F
CRC26	M	71	Transverse colon	ADC with mucinous component	pT2 N0	I	NA	WT	NA	MLH1, PMS2 loss	NA	No	20	Alive, CRC-F

*All tumours were low grade.

ADC, adenocarcinoma; *Chemo*, Chemotherapy; *CRC,* Colorectal cancer*; CRC-F* Colorectal cancer-disease free (as of last follow-up)*; FU,* Follow-up*; MSI,* Microsatellite instability; *MSS,* Microsatellite stable, *MSI-L,* Microsatellite instability-low; *MSI-H,* Microsatellite instability-high; *mo*, months; *NA* Not applicable; *NOS,* Not otherwise specified; *OC* Other causes; *PO* Post-operative complications; *WT* wild type; *Y* years, *(+)* overexpressed.
